# Causes of death among undocumented migrants in Sweden, 1997–2010

**DOI:** 10.3402/gha.v7.24464

**Published:** 2014-06-03

**Authors:** Anna Wahlberg, Carina Källestål, AnnaCarin Lundgren, Birgitta Essén

**Affiliations:** Department of Women’s and Children’s Health, International Maternal and Child Health (IMCH), Uppsala University, Uppsala, Sweden

**Keywords:** cause of death register, ICD-10, irregular migrants, death certificate, health care access

## Abstract

**Background:**

Undocumented migrants are one of the most vulnerable groups in Swedish society, where they generally suffer from poor health and limited health care access. Due to their irregular status, such migrants are an under-researched group and are not included in the country’s Cause of Death Register (CDR).

**Objective:**

To determine the causes of death among undocumented migrants in Sweden and to ascertain whether there are patterns in causes of death that differ between residents and undocumented migrants.

**Design:**

This is a cross-sectional study of death certificates issued from 1997 to 2010 but never included in the CDR from which we established our study sample of undocumented migrants. As age adjustments could not be performed due to lack of data, comparisons between residents and undocumented migrants were made at specific age intervals, based on the study sample’s mean age at death±a half standard deviation.

**Results:**

Out of 7,925 individuals surveyed, 860 were classified as likely to have been undocumented migrants. External causes (49.8%) were the most frequent cause of death, followed by circulatory system diseases, and then neoplasms. Undocumented migrants had a statistically significant increased risk of dying from external causes (odds ratio [OR] 3.57, 95% confidence interval [CI]: 2.83–4.52) and circulatory system diseases (OR 2.20, 95% CI: 1.73–2.82) compared to residents, and a lower risk of dying from neoplasms (OR 0.07, 95% CI: 0.04–0.14).

**Conclusions:**

We believe our study is the first to determine national figures on causes of death of undocumented migrants. We found inequity in health as substantial differences in causes of death between undocumented migrants and residents were seen. Legal ambiguities regarding health care provision must be addressed if equity in health is to be achieved in a country otherwise known for its universal health coverage.

Estimates of undocumented migrants in the European Union (EU) range from 1.9 to 3.8 million. Indications of a declining number are thought to be a result of the EU’s growth and its legalization programs (1). The corresponding figure for Sweden is estimated at 10,000–50,000, of which 2,000–3,000 are believed to be children (2). ‘Irregular migrants’, ‘unauthorized migrants’, ‘illegal migrants’, and ‘illegal aliens’ are all terms used to describe this group of people. We have chosen ‘undocumented migrants’, and defined it as follows: individuals who have entered the country illegally and never claimed asylum; asylum seekers who have been rejected and gone underground to avoid deportation; those who have overstayed their visa or work permit; EU citizens who are not compliant with regulations governing the right to residence (3).

Insecure working and living conditions among undocumented migrants are associated with psychological issues and somatic symptoms (4). Poor access to health care is one of the main issues for undocumented migrants. Its outcome is deteriorating health. Several barriers to health care access have been identified: absence of legal entitlements to health care, poor knowledge of a person’s right to health care, lack of financial resources, fear of being reported to the police or immigration authorities when seeking health care, administrative obstacles, and cultural barriers (5, 6).

Ambiguities in how to interpret legislation and a lack of guidelines complicate encounters between health care providers or social workers and undocumented migrants (7–9). Sweden has a universal health care system that covers all residents and is financed by taxes. However, the entitlement of undocumented migrants to health care is highly restricted (10). Since July 2013, undocumented migrants have had the right to subsidized care for conditions that require urgent medical attention, and undocumented children have the same rights as Swedish residents (11). Before that, they were entitled to unsubsidized emergency care only, except for former asylum-seeking children, who had the same rights as Swedish resident (12).

Due to their irregular status, undocumented migrants are an under-researched group and they are usually not included in national statistics (4). This is true in the case of the Swedish Cause of Death Register (CDR), a record maintained by the Swedish National Board of Health and Welfare (SNBHW). The CDR records the deaths of all Swedish residents, whether or not the person was a citizen or was present in Sweden at the time of death. However, those who are undocumented migrants, who died while seeking asylum or visiting Sweden, stillbirths, Swedish emigrants, and delayed death certificates of Swedish residents are not included (13). Thus, causes of death among undocumented migrants in Sweden are, to the best of our knowledge, unknown.

The primary aim of this study was to determine the underlying causes of death among undocumented migrants in Sweden by means of a previous unexploited source of data. A second objective was to establish whether there may be patterns in causes of death that differ between Swedish residents and undocumented migrants.

## Methods

### Study sample

This is a cross-sectional study of death certificates issued from 1997 to 2010 that were not included in the Swedish CDR. Copies of death certificates were obtained from the SNBHW and the Swedish National Board of Forensic Medicine (SNBFM).

**Fig. 1:**
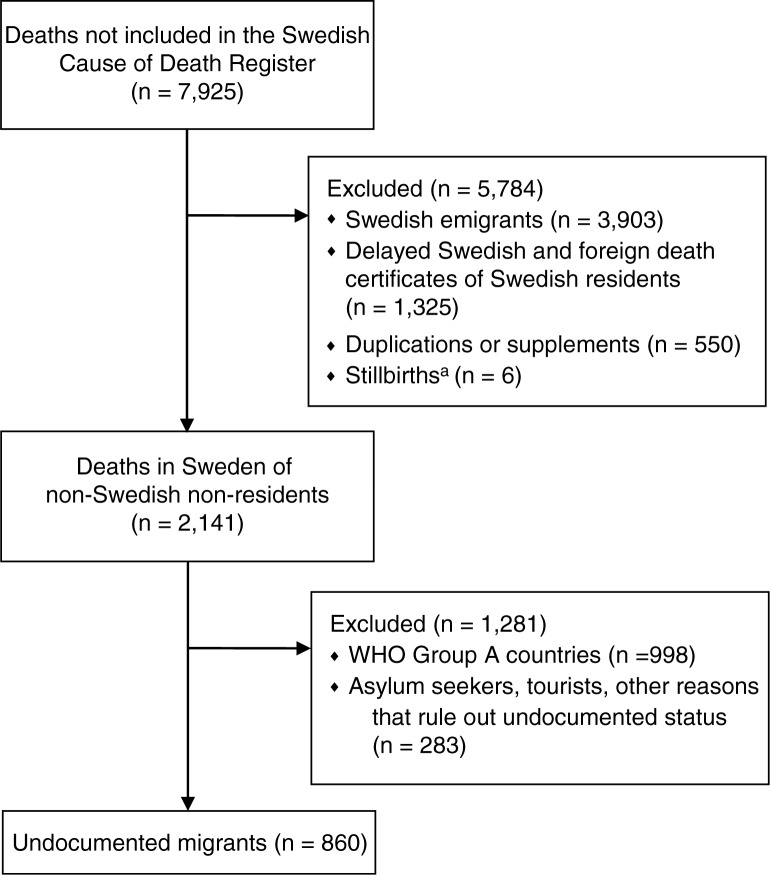
How the study sample of undocumented migrants was identified. ^a^Death certificates of stillbirths are not collected by the Swedish National Board of Health and Welfare, but the Swedish National Board of Forensic Medicine had *n*=6.

Our first task was to extract the study sample of undocumented migrants from the whole group of deaths not included in the CDR (Fig. 1). We excluded Swedish emigrants (death certificates of Swedish emigrants are often sent back to Sweden even though the deceased are no longer Swedish residents, or the Swedish emigrant dies while in Sweden on a visit), as well as delayed Swedish and foreign death certificates of Swedish residents (sometimes it takes years for the death certificate to reach the SNBHW, making it unfeasible to include it in the yearly statistics), duplicates or supplements, and stillbirths. To be identified as a Swedish resident or emigrant and subsequently excluded, at least two of the following pieces of information had to be present in the death certificate: a Swedish national identification number, a Swedish address, or a name of Swedish origin. People were also excluded if they were listed in the Swedish Death Index, a register of most deaths of residents that occurred in Sweden between 1901 and 2009, which includes data on emigrants. In addition, people from countries classified by WHO as group A (Table 1) (14), indicating that their child and adult mortality strata is very low, were excluded. We also made case-by-case assessments so that if information on a death certificate ruled out an undocumented migrant, for example truck drivers passing through Sweden, that person was excluded. If we thought someone might be an undocumented migrant, that individual was included. Thus, if nothing was known about the deceased except the cause of death, the person was included.

**Table 1:** Group A^a^ countries according to WHO classification of mortality stratum (14)

Andorra	Italy
Australia	Japan
Austria	Luxembourg
Belgium	Malta
Brunei Darussalam	Monaco
Canada	Netherlands
Croatia	New Zealand
Cuba	Norway
Czech Republic	Portugal
Denmark	San Marino
Finland	Singapore
France	Slovenia
Germany	Spain
Greece	Sweden
Iceland	Switzerland
Ireland	United Kingdom
Israel	USA

a Signifies very low child and adult mortality.

### Variables

The primary outcome was underlying cause of death, coded according to the International Classification of Diseases (ICD), 10th revision (15), using Iris version 4.2.0, the same software that the SNBHW employs for the CDR. Sex was categorized as male or female when so stated on the death certificate, or when it could be determined due to a gender-specific disease or a person’s name. Age at death was calculated whenever the dates of birth and death were known. If available, the deceased person’s country of origin was noted and classified by WHO geographical region (14). If the country of origin was not stated in the death certificate, classification into WHO geographical region was attempted based on the name of the deceased. The place of death was dichotomized as ‘death at hospital’ (including ambulance) or ‘death outside hospital’. We also indicated whether or not an autopsy was performed.

### Statistical analysis

Descriptive statistics were performed using SPSS for Windows (version 20.0). Statistical comparison between Swedish residents and undocumented migrants was performed for three age ranges, based on the study sample’s mean age at death±a half standard deviation: total sample 43.7±9.6 years (34–53 years), males 41.0±8.4 years (33–49 years), and females 49.7±11.1 years (39–61 years). A half standard deviation, rather than a whole, was chosen so that the age groups analyzed would not be too large. Age adjustments could not be made because the age composition of the living population of the study sample (the denominator population) was not known. Thus, by analyzing patterns of causes of death for a specific age interval, differences in causes of death due to different age composition in the groups compared are minimized. Cases with unknown age, sex, and cause of death were removed from the analysis of males and females. Cases with unknown age and cause of death were removed from the analysis of the total. Data for the comparison group, Swedish residents, were obtained from the CDR (13). Two-by-two tables in Epi Info (version 7.0.9.34) were used to calculate *p*-values using Pearson’s Chi-square test with Yates’ continuity correction, and odds ratios (OR) with 95% confidence intervals (CI). A *p*-value<0.05 was considered statistically significant.

### Ethical considerations

According to Swedish law, no ethics approval is needed for a study of deceased individuals. Permission was obtained from legal counsel for the SNBHW and the SNBFM to access the death certificates used in this study (3276–2012 and X13–90076).

## Results

### Characteristics of the study sample

In total, 860 deaths were classified as likely to have been those of undocumented migrants; the majority were males. Europe was the dominant point of origin, and there was great diversity in countries of origin (*n=*45). A total of 271 (33.3%) deaths occurred in a hospital, and 654 (77.3%) were autopsied (Table 2).

**Table 2:** Characteristics of study population of undocumented migrants (n=860) in Sweden, 1997–2010

		Male		Female		Total^a^	
				
		*n*	%	*n*	%	*n*	%
Sex	Determinable	614	75.1	204	24.9	818	100.0
	Missing data					42	4.9
Country of origin	Poland	140	48.3	28	30.8	171	43.3
	Lithuania	24	8.3	3	3.3	27	6.8
	Russian Federation	19	6.6	6	6.6	25	6.3
	Estonia	13	4.5	3	3.3	17	4.3
	Former Yugoslavia	10	3.4	4	4.4	16	4.1
	Others	84	29.0	47	51.6	139	35.2
	Total^b^	290	100.0	91	100.0	395	100.0
	Missing data					465	54.1
Region of origin^c^	Europe	404	74.5	113	63.1	532	71.2
	Eastern Mediterranean	72	13.3	27	15.1	104	13.9
	Africa	22	4.1	12	6.7	37	5.0
	America	20	3.7	14	7.8	35	4.7
	South-east Asia	14	2.6	9	5.0	25	3.3
	Western Pacific	10	1.8	4	2.2	14	1.9
	Total	542	100.0	179	100.0	747	100.0
	Missing data					113	13.1
Death at hospital	Yes	151	26.0	97	48.7	271	33.3
	Total	581	100.0	199	100.0	815	100.0
	Missing data					45	5.2
Autopsy	Yes	520	85.5	116	58.9	654	77.3
	Total	608	100.0	197	100.0	846	100.0
	Missing data					14	1.6
Age at death		*n*	Yrs.	*n*	Yrs.	*n*	Yrs.
	Mean	570	41.0	186	49.7	789	43.7
	Std. deviation		16.8		22.1		19.2
	Missing data					71	

a Total includes cases where the sex was unknown.

b Countries of origin present in this sample: *n*=45.

c 40.9% is based on origin of deceased’s name. Countries were classified in accordance with WHO regions (14).

### Underlying causes of death

As seen in Table 3, external causes of mortality (including suicide) were the most frequent underlying causes of death, accounting for 423 (49.8%) of all deaths among undocumented migrants. Dying from an external cause was more frequent among males (55.6%) than females (36.8%). Within the group of external causes, transportation accidents were the single largest cause of death (*n=*122), with 96 (28.3%) deaths among males and 23 (30.7%) deaths among females. Intentional self-harm (suicide) accounted for 92 (21.7%) deaths, 66 (19.5%) among males and 24 (32.0%) among females. The frequency of deaths from assault was 13.7% (*n=*58), with a slightly higher rate seen among males (14.2%) than females (10.7%) (Table 4).

**Table 3:** Frequency distribution of underlying causes of death among undocumented migrants, 1997–2010

	Male		Female		Total^a^	
			
Causes of death according to ICD-10	*n*	%	*n*	%	*n*	%
External causes of mortality	339	55.6	75	36.8	423	49.8
Diseases of the circulatory system	183	30.0	67	32.8	265	31.2
Neoplasms	23	3.8	23	11.3	52	6.1
Symptoms, signs, and abnormal findings	16	2.6	6	2.9	24	2.8
Diseases of the respiratory system	9	1.5	7	3.4	16	1.9
Diseases of the digestive system	9	1.5	4	2.0	13	1.5
Infectious and parasitic diseases	9	1.5	9	4.4	20	2.4
Endocrine, nutritional, and metabolic diseases	6	1.0	5	2.5	12	1.4
Diseases of the nervous system, eye, and ear	6	1.0	2	1.0	8	0.9
Mental and behavioral disorders	4	0.7	–	–	4	0.5
Congenital abnormalities	2	0.3	–	–	2	0.2
Conditions originating in the perinatal period	2	0.3	1	0.5	4	0.5
Diseases of the blood and immunity disorders	1	0.2	1	0.5	2	0.2
Diseases of the genitourinary system	–	–	3	1.5	3	0.4
Diseases of the skin and subcutaneous tissue	1	0.2	–	–	1	0.1
Pregnancy and childbirth	–	–	1	0.5	1	0.1
Total^b^	610	100.0	204	100.0	850	100.0
Missing data					10	1.2

a Total includes cases where the sex was unknown.

b One diagnostic group (diseases of the musculoskeletal system and connective tissue) had no deaths.

**Table 4:** Frequency distribution of ICD-10 category ‘external causes of mortality’ among undocumented migrants, 1997–2010

	Male		Female		Total^a^	
			
	*n*	%	*n*	%	*n*	%
Accidents						
Transportation accidents	96	28.3	23	30.7	122	28.8
Other accidental injuries^b^	123	36.3	17	22.7	142	33.6
Intentional self-harm	66	19.5	24	32.0	92	21.7
Assault	48	14.2	8	10.7	58	13.7
Other external causes^c^	6	1.8	3	4.0	9	2.1
Total	339	100.0	75	100.0	423	100.0

a Total includes cases where the sex was unknown.

b ICD-10 code W00–X59, for example, falls, drowning, exposure to fire.

c ICD-10 code Y10–Y98.

Diseases of the circulatory system (i.e. acute myocardial infarction, chronic ischemic heart disease, intracerebral hemorrhage, etc.) were the second most common cause of death among undocumented migrants, followed by neoplasms, both being more frequent among women than men. One pregnancy-related cause of death was found (Table 3).

### Comparison between Swedish residents and undocumented migrants

**Fig. 2:**
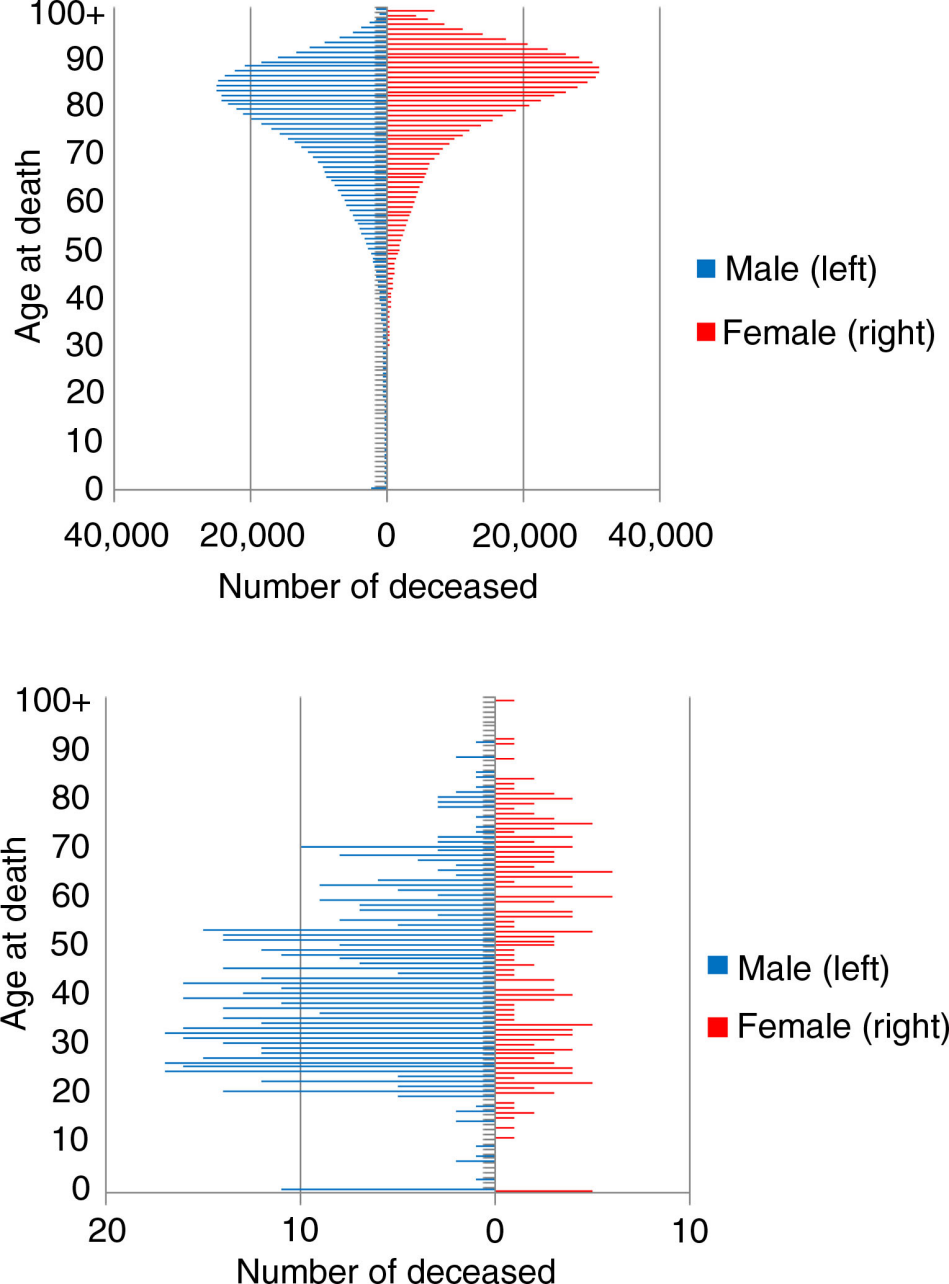
Age at death among Swedish residents^a^ (upper) and undocumented migrants (lower), 1997–2010. ^a^Data obtained from CDR (13).

Swedish residents and undocumented migrants were compared to determine if there are patterns in causes of death that differ between them (Table 5). The two groups had different age compositions. Mean age at death was much lower for undocumented migrants (see Fig. 2). Since age adjustments could not be made, analysis was carried out for specific age intervals to minimize the effect age structure might have had on cause of death (see Method section). Due to these limitations, statistical analyses were only performed on the three most common causes of death: external causes of mortality, diseases of the circulatory system, and neoplasms.

**Table 5:** Comparison of number of causes of death among Swedish residents and undocumented migrants, 1997–2010

	Males 33–49 years		Females 39–61 years		Total 34–53 years	
			
Causes of death according to ICD-10	Swedish residents^a^	Undocumented migrants	Swedish residents^a^	Undocumented migrants	Swedish residents^a^	Undocumented migrants
External causes (*n*)	7,825	121	4,568	17	13,226	150
OR (95% CI)	1	2.72 (2.04–3.61)	1	4.29 (2.41–7.64)	1	3.57 (2.83–4.52)
Circulatory system (*n*)	4,448	61	7,437	23	10,801	99
OR (95% CI)	1	1.71 (1.26–2.31)	1	3.98 (2.31–6.85)	1	2.20 (1.73–2.82)
Neoplasms (*n*)	4,377	3	25,088	7	18,462	10
OR (95% CI)	1	0.06 (0.02–0.19)	1	0.13 (0.06–0.28)	1	0.07 (0.04–0.14)
Total^b^ (*n*)	21,876	201	46,037	53	54,823	282

a Data obtained from CDR (13).

b Total includes all causes of deaths within specified age range.

All *p*-values from the Chi-square tests were <0.001.

Cases with unknown age, sex, and cause of death were removed from the analysis of males and females. Cases with unknown age and cause of death were removed from the analysis of the total. The number of missing cases in the whole study sample was: age *n=*71, sex *n*=42, and cause of death *n*=10.

Analysis was performed on mean age at death±a half standard deviation.

Undocumented migrants had a significantly higher risk of death from external causes (including suicide) compared to Swedish residents (OR 3.57, 95% CI: 2.83–4.52, *p<*0.001). Undocumented females were shown to have had a four-fold increased risk (OR 4.29, 95% CI: 2.41–7.64, *p<*0.001), and an increased risk could also be seen among undocumented males (OR 2.72, 95% CI: 2.04–3.61, *p<*0.001).

The risk of dying from diseases of the circulatory system was significantly higher among undocumented migrants (OR 2.20, 95% CI: 1.73–2.82, *p<*0.001), and undocumented females had the highest OR (OR 3.98, 95% CI: 2.31–6.85, *p<*0.001). Further, the risk of dying from neoplasms was significantly lower (OR 0.07, 95% CI: 0.04–0.14, *p<*0.001), with the lowest risk found among undocumented males who had an OR of 0.06 (95% CI: 0.02–0.19, *p<*0.001).

## Discussion

We identified 860 deaths in Sweden between 1997 and 2010 that may have been undocumented migrants. The most frequent causes of death were external ones (including suicide), followed by diseases of the circulatory system. Undocumented migrants were shown to have had a significant increased risk of death from both of the above in comparison to Swedish residents, while their risk of dying from neoplasms was significantly lower.

Since morbidity and mortality studies of undocumented migrants in Sweden are rare, we mainly used studies of foreign-born people living in Sweden to discuss the study results. The number of males in the study sample who died from external causes was greater than females. This could be expected since it is known that males in Sweden die from external causes to a greater extent than females (13). However, female undocumented migrants aged 39–61 had a surprisingly high risk of dying from external causes when compared with female Swedish residents in the same age group. Fernbrant et al. have shown that foreign-born women have a higher risk of domestic violence compared to Swedish-born women (16), and Esscher et al. found that foreign-born women of reproductive age coming from high-income countries more often die of external causes than the corresponding group of Swedish-born women (17). Deaths from external causes may be reduced through preventive measures, just as suicides may be prevented by implementation of effective social protections (18).

The second most common cause of death was diseases of the circulatory system, which is the leading cause of death globally (19). We found high ORs of dying from diseases of the circulatory system in our study sample, particularly for females. This coincides with studies of foreign-born individuals living in Sweden who have a higher rate of death from circulatory system diseases, a higher risk of myocardial infarction and hospitalization due to heart failure; and women born in Finland and Eastern Europe have a higher risk of mortality from coronary heart disease, than Swedish-born women (20–23).

Finding the risk of dying from neoplasms significantly lower in the study sample than in Swedish residents was somewhat unexpected, since Sweden has the lowest cancer mortality in Europe (24). However, previous research regarding cancer risk among the foreign-born in Sweden shows both increased and decreased risks, depending on the type of cancer and the person’s country of origin (17, 25–27).

In a study of undocumented migrants in 11 European countries including Sweden, the most commonly diagnosed health problems were musculoskeletal, digestive, and psychological (28). Médecins Sans Frontières in Sweden found digestive, respiratory, and cardiovascular issues to be common among undocumented migrants (6). Other than cardiovascular diseases, this was not reflected in our findings on causes of death. However, a given health issue is not always what an individual will die of later on.

A striking finding was the high number of autopsies carried out among undocumented migrants (77.3%), as compared to the frequency of autopsies among Swedish residents. The latter has dropped from 50% in the 1970s to 12% in 2010 (13). One explanation for this vast difference may be the relatively low number of deaths at a hospital in the study sample and the high number from external causes such as are more likely to trigger an autopsy.

Inequity in health and social welfare were reflected in our findings as it revealed substantial differences in causes of death between undocumented migrants and Swedish residents. This may partly be due to the fact that undocumented migrants in Sweden have poor access to health care. Further, undocumented migrants in Sweden lack entitlement to enter the regular housing and labor market. Their housing and working conditions are therefore precarious. They are often forced to pay unreasonably high rents. Unable to afford the rent themselves, they often live in overcrowded conditions and sometimes lodge together with other marginalized groups, such as alcoholics, drug addicts, and drug pushers. Their employment opportunities are commonly of short duration, underpaid, and insecure (2, 6, 29). Undocumented migrants have a high risk of being exposed to violence (2). However, as they are in hiding from the authorities, they have no possibility of notifying the police if they have been subjected to violence (29). As living and working conditions are linked to aspects of life-quality, such as health, these factors could be possible explanations for the high number of external causes, including suicides, accidents, and assault, among undocumented migrants in Sweden. In order to improve equity in health, access to health care for all, together with actions to address the social determinants of health are needed (30).

### Strengths and limitations

This study is based on previously unexplored data. It covers the entire Swedish nation over an extensive time period, yielding unique information about underlying causes of death among one of the country’s most vulnerable groups. The methodological limitations of this study are mainly due to the irregular status of undocumented migrants and their exclusion from the CDR. The SNBHW is planning to include parts of the country’s non-resident group in the Swedish CDR (13).

Since analysing undocumented migrants was our objective, we had to identify this group within the overall population of non-residents. No method for doing so existed, and little or no information about their reasons for being in Sweden around the time of their death was present in the death certificates. The WHO division of countries into mortality strata was chosen as a tool to exclude those who were most unlikely to be undocumented migrants. Our reasoning was that those coming from countries with good health (group A) would be less likely to migrate outside of regular channels. Further, none of the group A countries were present in the list of counties from which most asylum seekers came to Sweden between 1984 and 2011 (31). This is significant because most undocumented migrants in Sweden are rejected asylum seekers (29). Since we generally included people we believed may have been undocumented migrants, the sample could be an overestimation, with some tourists, asylum seekers, and others like them included, thus skewing the results. This may be reflected in the high number of sudden deaths from external and cardiovascular causes in our sample, while deaths that might have been prevented with better medical care if the afflicted person had been documented are fewer. To better connect deaths with the daily lives of undocumented migrants (such as accidents related to poor working conditions), death certificates would have to provide more information than they presently do. Linking these certificates with medical records may be a way to further this investigation.

Assessing the underlying cause of death was sometimes difficult due to the poor quality of the information on the death certificates, although only 10 death certificates completely lacked information about cause of death. As our study sample consisted of a wide range of people from different countries and backgrounds, their deaths are most likely affected by a variety of risk factors. However, assessment of these was beyond the scope of this paper. Regarding the variable ‘region of origin’, about 41% of the identifications were based on the deceased’s name, which introduces a risk of misclassification, although using a name to make predictions about a region or country of origin has been used in other studies (32, 33). Some of the differences in causes of death between the groups we compared may have been due to different age composition rather than actual differences in cause of death patterns. Male adults, a group that is expected to have a high number of deaths from external causes, might have been overrepresented in the group of undocumented migrants. The estimates could, therefore, be skewed.

## Conclusions

Information on causes of death can assist in monitoring health trends and discovering health gaps. The diversity of the study sample indicates that there is no single solution that can address the issue of poor health among undocumented migrants. Sweden has ratified a number of international human rights treaties that include right of access to health care services, regardless of citizenship or migratory status (7). Solutions must address the social determinants of health and the legal ambiguities regarding the provision of health care in order to achieve equity in health in a country otherwise committed to universal health coverage.
